# Public satisfaction with the Ethiopian healthcare system: a mixed methods approach

**DOI:** 10.3389/fpubh.2024.1275233

**Published:** 2024-10-02

**Authors:** Genanew Kassie Getahun, Bemnet Dires Demissie, Semere Gebremariam Baraki

**Affiliations:** ^1^Menelik II Medical and Health Science College, Addis Ababa, Ethiopia; ^2^The Three Aid Foundation, Barneveld, Netherlands

**Keywords:** public satisfaction, social ecological model, healthcare system, Ethiopia, mixed method study

## Abstract

**Introduction:**

The satisfaction of the public with the healthcare system of Ethiopia is a crucial but unanswered question. This is an essential issue since recent trends indicate that the demographic and epidemiological makeup of the population is changing. Therefore, the aim of this study was to assess the overall satisfaction of the public with the Ethiopian healthcare system in Addis Ababa, Ethiopia, in 2022.

**Methods:**

A community-based convergent parallel mixed methods study was conducted. Bivariable and multivariable logistic regression analyses were used to determine the factors associated with public satisfaction. A 95% confidence interval along with a *p* < 0.05 was deemed sufficient to declare a significant association. For the qualitative component, we used thematic analysis.

**Results:**

The vast majority, 77.2% (95% CI: 76.18–78.22%), of respondents were dissatisfied with the existing healthcare system. Moreover, Community-Based Health Insurance (CBHI) enrollment (2.35; 95% CI: 1.32–4.19), poor linkage to social capital (0.46; 95% CI: 0.25–0.83), poor access to healthcare services (0.39; 95% CI: 0.21–0.76), and absence of satisfactory responses to complaints (0.11; 95% CI: 0.04–0.27) were significantly associated with public dissatisfaction.

**Conclusion:**

Public satisfaction with the Ethiopian healthcare system is notably low and is affected by various factors, including enrollment in the CBHI, linkage to social capital, accessibility to healthcare, and satisfactory response to issues. Therefore, the Ethiopian government should focus on expanding CBHI coverage, improving access to healthcare services, and developing an immediate compliant solution strategy.

## 1 Introduction

Public satisfaction is a fundamental indicator of healthcare service quality and serves as a means of assessing healthcare performance (1). Globally, healthcare service strategies are becoming more reliant on public satisfaction, which is considered a basic criterion for evaluating clinical efficiency and performance improvement (2). Accreditation and regulatory requirements from organizations such as the National Committee on Quality Assurance (NCQA) and the Joint Commission on Accreditation of Healthcare Organizations (JCAHO) prove the need for quantifiable public satisfaction data as a crucial component of performance improvement programs (3, 4).

Public satisfaction is a comprehensive element that reflects consumers’ perceptions and attitudes toward their overall experience with healthcare services (5). The WHO uses public satisfaction with the healthcare system as one of the metrics to evaluate the quality of healthcare services (6–8). It is well recognized that patient satisfaction significantly influences their likelihood of seeking additional medical advice, adhering to their treatment plan, and maintaining an ongoing and friendly relationship with their healthcare practitioner (9, 10). In addition, public satisfaction might vary depending on the tool used to measure it, making it difficult to determine a precise level of satisfaction (11, 12). A study carried out in 30 different countries found that, despite some variations within each of those countries, people in the developed world generally agree that public acceptance is fairly high (8).

While accessibility, quality, financial burden, and healthcare equity have all been individually measured and assessed in the past, in countries such as Ethiopia, public opinion can only be inferred from other studies in which patient and/or healthcare professional satisfaction has been fairly explored (13, 14). The evaluation of public perception and satisfaction might aid in the decision-making process to improve the responsiveness of the healthcare system (15–17).

The Ethiopian healthcare system is vulnerable to many criticisms and opinions (18). Issues related to poor healthcare delivery, minimal healthcare coverage, and low adherence to community-based insurance are just a few of the numerous factors that could be taken into consideration (19, 20).

The assessment of public opinion and satisfaction can help for a better understanding of the public’s needs and improve quality health services by identifying their determinant factors through a continuous quality improvement process (21, 22). Ignorance of public opinion, on the other hand, may be a roadblock to reaching milestones by combining public perception and understanding with available resources to achieve universal healthcare (17, 23). In addition, in the context of Ethiopia’s current health sector reform, healthcare facilities are working to improve the effectiveness, efficiency, and quality of the services they provide (24). Moreover, this study was conducted to explore the current context and the determinants of healthy individuals’ satisfaction with the health services provided after the reform. Therefore, this study aimed to assess the overall satisfaction of healthy individuals with the Ethiopian healthcare system in Addis Ababa, Ethiopia, in 2022.

## 2 Methods

### 2.1 Study area and period

This study was conducted in Addis Ababa, Ethiopia. Addis Ababa is the capital city of Ethiopia, which is a metropolitan city in the highlands of Ethiopia with an area of 527 km^2^, an elevation of 2,355 m^2^, and a population of approximately 5 million people (25). Addis Ababa is the most densely populated and urbanized city in Ethiopia, with a high concentration of both public and private health institutions and an easily accessible target group. There are 11 sub-cities and 116 districts. The data were collected between February and April 2022.

### 2.2 Study design and population

We used a community-based convergent parallel mixed methods study (interpretative paradigm; a social science approach that asserts that understanding the beliefs, motivations, and reasoning of individuals in a social situation is critical to decoding the meaning of data gathered around a phenomenon). All households in selected sub-cities of Addis Ababa, Ethiopia, were considered the study population.

### 2.3 Eligibility criteria

All selected individuals over the age of 18 years were included in the study. However, individuals who lived in the selected areas for <6 months or who were unable to communicate for any reason were excluded from the study.

### 2.4 Sample size determination and sampling procedure

For the quantitative study, the sample size was calculated using a single population proportion formula considering an assumption of a 50% prevalence to have a relatively larger sample size, a 5% precision at the 95% confidence level, and a 15% non-response rate.

$n=\frac{\frac{{\left(Z\alpha 1\right)}^{2}}{2}P\left(1-P\right)}{d^{2}}$

Since multistage sampling was used, considering a 1.5 design effect and a 15% non-response rate provided a required sample size of 663. For the qualitative study, a theoretical maximum saturation technique was applied.

Multistage sampling was used, starting with the 11 sub-cities of Addis Ababa. Simple random sampling (lottery method) was used to select three sub-cities (Kolfe Keranio, Yeka, and Lideta). From each sub-city, three districts (woreda) were chosen using the lottery method, and using proportional allocation, the number of households was determined.

A convenience sampling method was used for the key informant interviews. New volunteers were gathered for the interview until data saturation was achieved (no new ideas were emerging). Subsequently, nine participants were included in this study. The goal of the qualitative components was to explain the barriers and facilitators of public satisfaction.

### 2.5 Study variables and operational definition

Public satisfaction was considered an outcome variable, and the independent variables were categorized as intra-personal, interpersonal, community, and organizational-related factors.

*Intra-personal factors were as follows:* age, sex, educational status, employment status, income, health status, and wellbeing.

*Interpersonal factors were as follows:* social capital (bonding, bridging, and linking), marital status, and family size.

*Organizational and public policy* included political affiliation, enrollment in a health insurance scheme, and attitude toward the healthcare system.

*Satisfaction factors* included fulfillment of one’s objectives, expectations, or requirements in relation to one’s healthcare experience.

*Healthcare system* included institutions, people, and resources involved in providing public healthcare.

### 2.6 Data collection procedure

The data were collected using a pretested questionnaire, which was developed after reviewing previous similar studies (9, 11, 14). For the quantitative part, data were collected through an interviewer-administered questionnaire that was developed in English first and translated into the local language Amharic and then returned to English to ensure consistency and maintain conceptual equivalence. The data were collected through house-to-house visits. The data collection was begun with the permission and consent of the study participants. The respondents were quizzed on socio-demographic, interpersonal or relationship, organizational, and policy-related questions. The validity of the questionnaire was evaluated by subject-matter experts and senior researchers. Moreover, the reliability of the tool was checked using Cronbach’s alpha coefficient, and the result was 0.71, indicating that the rule was not violated. Data quality was assured through pretesting the questionnaire and careful supervision during data collection. Pre-testing was conducted at Arada Sub-City with 34 study participants prior to the actual data collection to estimate the time required for each data collection and testing the tool. Following that, we made modifications to some components of the questionnaire.

The qualitative data, on the other hand, were collected through an in-depth discussion guided by an interview guide. The interview guide asked questions about the importance of friends, family, and society in making decisions about healthcare, the quality and expense of healthcare services, the attitudes, behaviors, and communication styles of healthcare providers, the accessibility of medical supplies and resources through community-based health insurance programs, and the respondents’ satisfaction with the overall services received.

All of the interviewees provided their consent for their voices to be recorded and interviewed. The interview lasted between 30 and 45 min. The key informants that were approached for this study had no prior relationship with the interviewer, which helped to minimize the possibility of bias.

### 2.7 Data quality management and analysis

Before analysis, the data were cleansed and double-checked for completeness and consistency. The quantitative data were collected, categorized, coded, and entered into EPI Info version 7.2.1 before being exported to SPSS for further analysis. A binary and multivariable logistic regression analysis was performed to assess the statistical significance and strength of the association between independent variables and public satisfaction. A *p <* 0.05 in the final multivariable logistic regression analysis model was considered significantly associated. Based on the data acquired, the findings of the study were provided in the form of tables, figures, and text. The results of the study were presented using tables, figures, and texts.

For the qualitative component, we used an inductive technique to investigate the intricacies of the public’s perspective of the healthcare system, as well as the reasons for their contentment or displeasure with it. Data were collected only from adults since they were more likely to interact with the healthcare system on their own. The interviews were transcribed for analysis by the principal investigators, and the remaining two co-authors, who are skilled in collecting qualitative data, translated the transcripts into English. Then, we looked for recurring themes and variances in the transcripts and field notes. After that, the coder read over each transcript to make sense. Using the preliminary code guide, all transcripts were coded individually. The final version of the code guide was developed by following these procedures. Then, the transcript was converted to open-code 4.03 format. Finally, the investigators looked into the relationships between the codes that had been generated previously and categorized them into groups.

### 2.8 Ethical consideration

Ethical clearance was obtained from Kotebe Metropolitan University, Menelik II Medical and Health Science College Ethical Committee. A permission letter was secured from the Addis Ababa City Administration Health Bureau Public Health Record and Emergency Management Directorate. Formal letters of cooperation were given to each district, and written consent was obtained from individual participants by explaining the aim of the research.

## 3 Results

### 3.1 Socio-demographic characteristics of the study participants

From a total of 663 study participants, a complete set of information was obtained from 649 respondents, giving a response rate of 97.9%. The mean and standard deviation of the age of respondents were 41.3 (±15.8) years, with a range of 18–86 years. The majority of the study participants were private workers and employees, comprising 26.8 and 24.3%, respectively. More than half (53.6%) of the respondents were women (Table 1).

**Table 1:** Socio-demographic characteristics of the study participants.

Variable	Category	Frequency	Percentage
Age	Young adults 18–30	201	30.9
	Adult 30–60	374	57.7
	Older adult >60	74	11.4
Sex	Male	301	46.4
	Female	348	53.6
Educational status	None	64	9.9
	Primary school	104	16
	Secondary school	181	27.9
	Diploma	98	15.1
	Bachelor’s degree	158	24.3
	Postgraduate and above	44	6.8
Occupation	Public servant	108	16.6
	Private employee	158	24.3
	Private work	174	26.8
	Pensioner	60	9.2
	Student	20	3.1
	Unemployed	41	6.3
	Housewife	88	13.6
Health status (personal opinion)	Poor	82	12.6
	Fair	77	11.9
	Good	169	26
	Very good	198	30.5
	Excellent	123	19

### 3.2 Interpersonal (relationship)-related characteristics

According to the data obtained from this study, the majority (66.4%) of the study participants reported that they had a strong linking social capital followed by 66.1% bonding social capital as a relationship-related domain (Table 2).

**Table 2:** Results for the interpersonal domain.

Variable	Response	Frequency	Percentage
Bonding social capital	Yes	429	66.1
	No	220	33.9
Bridging social capital	Yes	381	58.7
	No	268	41.3
Linking social capital	Yes	218	33.6
	No	431	66.4
Types of support	Information	116	52.25
	Money	20	9
	Enabling access	76	34.2
	Other	10	4.6
Believe it is right to use LSC	Yes	175	29.7
	No	414	70.3

### 3.3 Organizational and public policy-related domains

Almost all (95.5%) respondents reported having visited health institutions, with public health organizations being the most common. In addition, only 40.7% of the respondents believed that they had emergency access to health services. Moreover, 25.8 and 10.7% of the total individuals interviewed were CBHI users and satisfied with the responses provided by healthcare providers, respectively (Table 3).

**Table 3:** Results for the organizational and public policy domains.

Variable	Response	Frequency	Percentage
Visit a health institution	Yes	620	95.5
	No	29	4.5
Types of health services used	Public	402	61.9
	Private	119	18.3
	Both	128	19.7
Access	Yes	440	67.8
	No	209	32.2
chronic illness	Yes	169	26.0
	No	480	74.0
Emergency access	Yes	264	40.7
	No	385	59.3
No treatment due to cost	Yes	281	43.3
	No	368	56.7
Effect of cost on the HH economy	Yes	463	71.3
	No	186	28.7
Health insurance	Yes	164	25.3
	No	485	74.7
CBHI awareness	Yes	389	59.9
	No	260	40.1
CBHI use	Yes	114	25.8
	No	327	74.2
CBHI beneficial	Yes	364	82.5
	No	77	17.5
Expressed complaints to staff	Yes	215	33.1
	No	434	66.9
If not, why	I assumed no one would listen	91	27
	I assumed my complaint would not be solved	80	23.6
	My own negligence	167	49.4
	Other	96	
I received a satisfactory response	Yes	69	10.6
	No	149	23.0

### 3.4 Level of public satisfaction with the Ethiopian healthcare system

**Figure 1:**
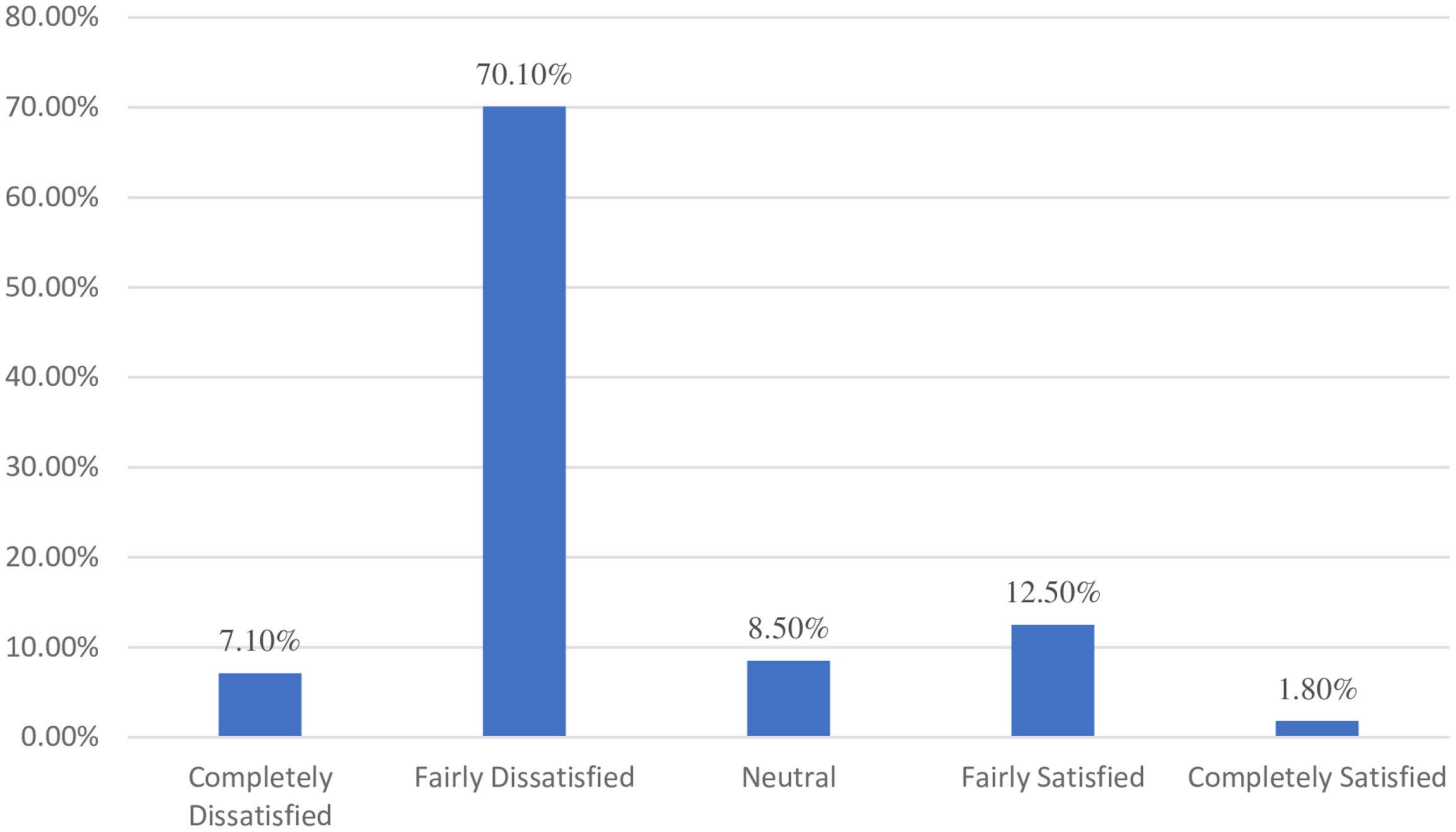
Level of satisfaction among respondents.

The majority of respondents, 77.2% (95% CI: 76.18–78.22), were dissatisfied with the existing healthcare system, with 7.1% being completely dissatisfied. On the other hand, 12.5% reported being fairly satisfied, and 1.8% responded that they were completely satisfied (Figure 1).

### 3.5 Factors associated with respondents satisfaction level

In multivariable logistic regression analysis, variables with a *p* < 0.25 from the bivariable analysis were identified. As a result, CBHI enrollment, linking with social capital, access to healthcare services, and satisfactory responses to client complaints were significantly associated with the satisfaction level at a *p* < 0.05.

Respondents enrolled in CBHI had more than two times (2.35, 95% CI: 1.32–4.19) more odds of being satisfied with the Ethiopian healthcare system than their counterparts who were not enrolled. On the other hand, study participants who had poor linkage to social capital had (0.46; 95% CI: 0.25–0.83) 54% higher odds of being dissatisfied with the Ethiopian healthcare system than their counterparts. Furthermore, study participants who had unsatisfactory responses to their complaints had 0.11 (95% CI: 0.04–0.27) or 89% more odds of having poor satisfaction than their counterparts. Study participants who had poor access to health care services had a 61% higher odds of dissatisfaction 0.39 (95% CI: 0.21-0.76) compared with their counterparts (Table 4).

**Table 4:** Factors associated with satisfaction in the Ethiopian healthcare system.

Variable	Satisfaction		Odd ratio	
	Satisfied	Unsatisfied	COR(95% CI)	AOR(95% CI)
Sex				
Male	54	247	1.79(1.13–2.83)	1.21(0.37–3.93)
Female	94	254	1	1
CBHI use				
Yes	64	94	3.29(2.29–4.91)	2.35(1.32–4.19)**
No	84	407	1	1
Poor linking to social capital				
Yes	63	314	0.44(0.24–0.0.73)	0.46(0.25–0.83) *
No	85	187	1	1
Poor access to healthcare				
Yes	115	325	2.18(1.27–3.75)	0.39(0.21–0.76)*
No	33	176	1	1
Health status				
Sick	40	119	0.72(0.61–0.85)	0.61(0.32–1.17)
Well	108	382	1	1
Unsatisfactory response				
Yes	41	28	0.12(0.06–0.23)	0.11(0.04–0.27) **
No	22	127	1	1

95% confidence interval: ^*^p < 0.05, ^**^p < 0.01.

### 3.6 Qualitative results

The respondents’ opinions can be categorized into three clusters or themes that cross over one another. The key finding is that respondents to varying degrees were dissatisfied.

#### 3.6.1 Theme 1: cost of healthcare

One of the main causes of respondents’ discontent with the healthcare system was mentioned as the expense of healthcare or financial obstacles. A 45-year-old government-employed male study participant explained the issue as follows:

“*In contrast to affordability and access, the primary concern of private health service providers is money. The cost makes it challenging to receive treatments on a regular basis. As I noticed it throughout the time of my surgery.”*

Other respondents agreed that CBHI plays a critical role and expressed hope that it would help ease some of the financial challenges that public healthcare facilities, particularly in the public sector, are currently experiencing. Two women, aged 51 and 39 years, expressed their opinions as follows:

*“I’ve heard about CBHI, and it sounds like a wonderful idea. Although I do not use CBHI, it would be beneficial for those with chronic illnesses like mine to have more affordable access to care and medication.”*

*“I am confident that CBHI offers treatment and aftercare services at a reasonable cost since I have seen it in action.*”

#### 3.6.2 Theme 2: attitudes and behaviors of health personnel

One major source of participant unhappiness was the attitude of the medical staff. People who have social connections with healthcare professionals tend to get better care, which is largely related to the interpersonal level of social capital linkage.

*“People who have social relationships with healthcare experts have better access to care, prescriptions, and other facilities in both the private and public health sectors. However, if you do not have someone, you will inevitably experience all the drawbacks, especially if you aren’t paired with a trustworthy or familiar person.”*

In addition, a widespread criticism among participants was the administrative staff’s and professionals’ failure to listen to and address issues. Moreover, a 35-year-old man reported his experience as follows:

*“Based on my experience, understanding patient concern is critically important to enhance health services, but doing so is impossible due to the unwillingness of administrative and medical staff to take complaints seriously or find solutions.”*

Another thing to note is that respondents have extremely strong ideas about how unqualified health service providers treat people, and several said they would stress adequate training for health workers if they could.

“*According to my observations, one of the major issues in the nation is the carelessness of professionals, which is a result of their inadequate training and education as well as their unethical treatment of patients. Because of the carelessness of specialists, I have lost relatives.”*

#### 3.6.3 Theme 3: public opinion on the adequateness of medical facilities and their services

According to previous findings, the public is dissatisfied with the healthcare system and the health services provided in both private and public settings for a variety of reasons. The majority of respondents cited the inadequacy of medical facilities and their services as one of the main causes of this dissatisfaction.

*“Despite the high cost, private health institutions provided relatively better satisfaction despite having insufficient access to both public and private healthcare facilities. My personal experience with the medical care I have received and witnessed being provided to others has left me very unsatisfied.”*

Some respondents stated that the major reasons they feel the need to attend private institutions are the difficulties they encounter and the general inadequacy of public institutions.

*“I avoid going to public health facilities because it’s difficult to find professionals there, and even when medications have been ordered, it can be challenging to obtain them. I’ve also seen people travel far and wide in search of medications that are out of their price range. For these reasons, I usually go to private health facilities. Going to a public health facility is usually inconvenient, which is why I select a private facility.”*

## 4 Discussion

This study used a mixed methods approach to provide an assessment of public opinions and satisfaction with the healthcare system in Ethiopia. The degree of satisfaction with the given healthcare services significantly influences a person’s decision to seek medical advice, adherence to treatment, and keeping a positive connection with healthcare providers. According to this report, dissatisfaction (77.2%; 95% CI: 76.18–78.22) with the healthcare system is far greater than satisfaction. It is higher than previous study findings from different parts of Ethiopia, including 53.8% in Addis Ababa (26), 38.1% in Southwest Ethiopia (27), 36.3% from a systematic review and meta-analysis in Ethiopia (28), and 33% in Nairobi, Kenya (29). The possible reason behind this might be due to the difference in study period and measurement tools used to quantify the satisfaction. In addition, the variations in the dedication of healthcare administrators and healthcare professionals may be the difference.

Several significant factors were observed in this study, with four standing out: CBHI enrollment, social capital linkage, access to healthcare services, and a satisfactory response from health professionals regarding their complaints were statistically associated with public satisfaction.

As established in the quantitative portion and supported by the qualitative interviews, participants who used and believed in their linking social capital with health professionals were more likely to be satisfied with the healthcare system. As a result, participants with poor linking social capital had 54% (95% CI: AOR = 0.46, 0.25–0.83) higher odds of dissatisfaction than their counterparts. This result was in line with a study finding from Ghana (14). According to the findings, people are more likely to receive better care when they have a social relationship with medical experts. This will consequently change their perception of the efficacy and caliber of the care they receive. On the other hand, people without a strong social network might feel that it is inappropriate to use and are considerably less likely to be happy with the medical care they receive. The qualitative findings also reveal that there is some animosity toward individuals who have social capital links and toward professionals who are believed to treat people with whom they have a social relationship more favorably (30, 31).

When compared to non-users, those who were enrolled through CBHI were substantially more likely to report feeling satisfied with the current state of healthcare. As a result, those research participants who used CBHI had more than two times (95% CI: AOR = 2.35, 1.32–4.19) higher odds of being satisfied than their counterparts. Studies previously carried out in Bangladesh and Ethiopia reported similar findings (32, 33). This could be due to the fact that people’s happiness with the health system is greatly influenced by the absence or reduced burden of health-related costs as these charges are more likely to have an impact on their household budgets (32). Even though the majority of the respondents in the qualitative component were not CBHI beneficiaries, it is noted that some respondents favor and think it should be made more freely available.

Moreover, the findings of this study showed that those study participants who have had their complaints satisfactorily resolved have a much higher likelihood of being pleased with their health treatment. The result was consistent with previous study results conducted in different countries (34–36). According to the in-depth interview findings, respondents’ major complaints were about appropriate and satisfactory responses from healthcare providers. It is important to emphasize that, in spite of every complaint that respondents have voiced, how the public perceives the healthcare system is mostly dependent on the competence of administrators, personnel, or healthcare professionals to appropriately resolve issues.

Poor access to healthcare services was also identified as a significant factor for public dissatisfaction. Those respondents who perceived poor access to healthcare services had 89% higher odds of dissatisfaction with the healthcare system than their counterparts. It was consistent with a study finding from earlier research (37, 38). The fact that healthcare services are becoming a more competitive market justifies the negative correlation between poor access to healthcare services and satisfaction with the care received.

## 5 Limitations of the study

The study was conducted from the viewpoint of the general public, as opposed to patients who had more recent experiences. As a result, this study may have some recall bias. Furthermore, because the study concentrated on the general population and broad socio-ecological issues, it was challenging to dig deeply into the specifics. Therefore, this could be a subject for future research including a larger and more diverse sample size.

## 6 Conclusion

According to the current study findings, the majority of respondents (77.2%) were dissatisfied with the Ethiopian healthcare system. Furthermore, the benefit of CBHI, access to healthcare services, and satisfactory resolutions of complaints were the most important elements for public satisfaction. As a result, managers of healthcare facilities should develop a strategy for improving access to healthcare services and CBHI enrollment to improve the satisfaction of the public with the Ethiopian healthcare system.

## Data Availability

The original contributions presented in the study are included in the article/supplementary material, further inquiries can be directed to the corresponding author.
